# Reversal of ABCG2-mediated drug resistance by tinodasertib (ETC-206)

**DOI:** 10.3389/fphar.2025.1606857

**Published:** 2025-06-13

**Authors:** Haigan Yang, Zheshen Li, Zhuoxun Wu, Xiang Chen, Letao Bo, Harsh Patel, Bohan Zhang, Wenjun Xiong, Wei Wang, Zhe-Sheng Chen

**Affiliations:** ^1^ Department of Pharmaceutical Sciences, College of Pharmacy and Health Sciences, St. John’s University, Queens, NY, United States; ^2^ The First Affiliated Hospital of Guangzhou University of Chinese Medicine, Guangzhou, Guangdong, China

**Keywords:** ATP-binding cassette (ABC) transporter, tinodasertib, ETC-206, ABCG2, multidrug resistance (MDR)

## Abstract

**Introduction:**

Multidrug resistance (MDR) in cancer therapy, frequently driven by overexpression of ATP-binding cassette (ABC) transporters—particularly ABCG2—continues to undermine the efficacy of chemotherapeutic regimens. Tinodasertib (ETC-206), a selective ATP-competitive MNK1/2 kinase inhibitor, currently in Phase II clinical trials, has not yet been evaluated for its capacity to counteract ABCG2-mediated drug efflux. This study investigates whether tinodasertib can reverse ABCG2-dependent MDR in both two-dimensional (monolayer) cancer cell cultures and three-dimensional multicellular tumor spheroids.

**Materials and Methods:**

ABCG2-overexpressing cancer cell lines and their nonresistant parental counterparts were cultured as monolayers and as multicellular spheroids. Cell viability analysis in the presence or absence of tinodasertib was performed by MTT assay. Western blotting and immunofluorescence studies assessed ABCG2 protein expression and subcellular localization following tinodasertib exposure. ATPase activity of purified ABCG2 was measured in the presence of increasing tinodasertib concentrations. *In silico* docking simulations were conducted to model tinodasertib binding to the ABCG2 transmembrane region.

**Results:**

In monolayer cultures, co-administration of tinodasertib significantly sensitized ABCG2-overexpressing cells to mitoxantrone and topotecan. Similar enhancement of cytotoxicity was observed in multicellular tumor spheroids, where tinodasertib reduced the spheroid growth when combined with ABCG2 substrates (p < 0.05). Western blot and immunofluorescence analyses showed no change in total ABCG2 protein levels or its membrane localization upon tinodasertib treatment. ATPase assays revealed a dose-dependent inhibition of ABCG2 ATP hydrolysis (IC_50_ ≈ 2 μM for ATPase activity). Docking studies predicted high-affinity binding of tinodasertib within the substrate-binding cavity of ABCG2, consistent with competitive inhibition of ATPase function.

**Discussion:**

These data indicate that tinodasertib effectively reverses ABCG2-mediated MDR by blocking the transporter’s ATPase-dependent efflux mechanism without altering ABCG2 expression or trafficking. The concordance between ATPase inhibition and *in silico* docking supports a model wherein tinodasertib occupies the substrate-binding pocket of ABCG2, preventing ATP hydrolysis and subsequent drug transport. Overall, combining tinodasertib with ABCG2 substrate chemotherapeutics may represent a promising strategy for overcoming MDR in tumors overexpressing ABCG2, warranting further *in vivo* validation and clinical evaluation.

## 1 Introduction

Cancer continues to be a primary cause of morbidity and mortality worldwide (Frick et al., 2023; Zaorsky et al., 2017), with drug resistance posing a major challenge to successful treatment (Roszkowska, 2024; Vasan et al., 2019). The overexpression of ATP-binding cassette (ABC) transporters represents a central mechanism driving multidrug resistance (MDR), with ATP-binding cassette subfamily G member 2 (ABCG2), commonly referred to as the breast cancer resistance protein (BCRP), playing a particularly crucial role (Wu et al., 2022; Austin Doyle and Ross, 2003; Kim et al., 2002). ABCG2 actively pumps chemotherapeutic agents out of cancer cells, reducing their intracellular concentrations and diminishing their cytotoxic efficacy (Kukal et al., 2021; Guo et al., 2018). The broad substrate specificity and high expression of ABCG2 in various malignancies highlight its clinical significance as a target for overcoming MDR (Dong et al., 2024; Wu et al., 2021a; Goracci et al., 2023).

The development of effective ABCG2 inhibitors has been hindered by challenges such as low specificity, high toxicity, and limited success in clinical applications (Kumar et al., 2025; Wu et al., 2023; Feyzizadeh et al., 2022). For example, Fumitremorgin C (FTC) and its analogs (e.g., Ko143), although widely used in preclinical studies due to their potent inhibition of ABCG2, are associated with neurotoxicity (Allen et al., 2002) and poor pharmacokinetic profiles, limiting their clinical applicability (Yu et al., 2024). This emphasizes the critical need for innovative compounds with improved safety and efficacy to regulate ABCG2 function. Tinodasertib (ETC-206) is a selective ATP-competitive inhibitor targeting MNK1/2 kinases, which play a crucial role in regulating mRNA translation through interaction with eIF4E. By suppressing MNK1/2 activity, Tinodasertib inhibits the synthesis of tumor-associated proteins, thereby reducing cancer cell proliferation and survival. In clinical trials, Tinodasertib is currently being evaluated for its safety, pharmacokinetics, and therapeutic potential, particularly in combination therapies for cancers like metastatic colorectal cancer (Teneggi et al., 2020).

Although tinodasertib shows promising effects as a transcriptional inhibitor and in inhibiting tumor progression, its potential to modulate MDR mechanisms has yet to be fully explored. ABCG2, a key transporter involved in MDR, expels chemotherapeutic drugs from cancer cells, thereby diminishing their effectiveness. Therefore, investigating how tinodasertib interacts with ABCG2 and whether it can overcome drug resistance could greatly enhance its clinical utility, especially in cancers where resistance to standard treatments poses a significant challenge.

In this study, we aim to elucidate the mechanistic role of tinodasertib in modulating ABCG2 function and its potential in reversing MDR. Through a combination of *in vitro* and *in silico* approaches, we investigated tinodasertib’s effects on ABCG2-mediated drug efflux, transporter expression, and ATPase activity. Furthermore, we assessed tinodasertib’s capacity to enhance the susceptibility of drug-resistant neoplastic cells to conventional chemotherapeutic agents. Our results seek to enrich the comprehension of its therapeutic implications, offering novel insights regarding its application as an adjunct in oncological interventions, especially for individuals afflicted with multidrug resistance-associated malignancies.

## 2 Materials and methods

### 2.1 Chemicals and reagents

Tinodasertib was sourced from ChemieTek (Indianapolis, IN, United States). Sigma-Aldrich (St. Louis, MO) supplied chemical agents such as Ko 143, 4′,6-diamidino-2-phenylindole (DAPI), cisplatin, topotecan, and mitoxantrone. Corning Inc. (Corning, NY) provided fetal bovine serum (FBS), Dulbecco’s modified Eagle’s medium (DMEM), bovine serum albumin (BSA), and 0.25% trypsin. A monoclonal human antibody targeting ABCG2 was procured from Millipore (Billerica, MA). Rabbit anti-mouse IgG secondary antibody conjugated with horseradish peroxidase (HRP) came from Cell Signaling Technology Inc. (Danvers, MA). Alexa Fluor 488-conjugated goat anti-mouse IgG secondary antibodies and GAPDH, used as a loading control, were purchased from Thermo Fisher Scientific Inc. (Rockford, IL). Moravek Biochemicals (Brea, CA) supplied [3H]-labeled mitoxantrone (2.5 Ci/mmol). All remaining chemicals were acquired from Sigma Co. (St. Louis, MO).

### 2.2 Cell lines and cell culture

The S1-M1-80 cell line, originating from human colon carcinoma S1 cells, was developed by prolonged selection in 80 µM mitoxantrone, resulting in the overexpression of ABCG2 (Cheng and To, 2012; de Bruin et al., 1999). Drug-resistant NCI-H460/TPT10 cells, overexpressing ABCG2, were developed by subjecting NCI-H460 cells to prolonged exposure to 10 nM topotecan (Lei et al., 2020). The NCI-H460-KO and NCI-H460/TPT10-KO cell lines, with ABCG2 gene knockout, were created using the CRISPR/Cas9 gene-editing system (Lei et al., 2020). HEK293 cells, stably transfected with either an empty pcDNA3.1 vector or a pcDNA3.1 vector encoding the full-length ABCG2 protein, were cultured in Dulbecco’s Modified Eagle’s Medium (DMEM) containing 10% fetal bovine serum (FBS). Transfected sublines were maintained by selection in a medium with 2 mg/mL G418 (Robey et al., 2003). All the aforementioned cell lines were grown in DMEM enriched with 10% FBS and 1% penicillin/streptomycin, under conditions of 37°C, 5% CO2, and high humidity. Before experimentation, the drug-resistant cell lines were cultured in a complete medium free of drugs for a minimum of 14 days.

### 2.3 Cell viability and reversal experiments

Cell viability and resistance fold were evaluated through the MTT assay, following established protocols (Ji et al., 2019). For the reversal experiment, cells were collected, resuspended, and evenly plated in a 96-well plate at a density of 5 × 10^3^ cells per well in 160 μL of culture medium. After 24 h of incubation, tinodasertib was introduced 2 hours before adding the anticancer agents. Following a 72-h treatment period, 4 mg/mL MTT solution was added to each well, and the plates were incubated for another 4 h. The supernatant was discarded, and 100 μL of DMSO was used to solubilize the formazan crystals. Absorbance at 570 nm was recorded using an accuSkan™ GO UV/Vis Microplate Spectrophotometer (Fisher Scientific, Fair Lawn, NJ). The IC50 values for the anticancer drugs were determined as previously described. (20) Ko 143 (3 μM) was included as a positive control to reverse ABCG2-associated multidrug resistance, while cisplatin, which is not an ABCG2 substrate, served as a negative control.

### 2.4 Western blotting analysis

Western blot analysis was conducted based on established methods (Chen et al., 2024). Proteins from cells treated with tinodasertib were isolated using RIPA lysis buffer containing 1% protease inhibitor cocktail (Sigma-Aldrich, St. Louis, MO). Protein concentrations were measured with a BCA assay kit and resolved by SDS-PAGE on precast polyacrylamide gels (Bio-Rad, Hercules, CA), followed by transfer to PVDF membranes (Millipore, Burlington, MA). The membranes were blocked using 5% non-fat milk and incubated overnight at 4°C with specific primary antibodies. Primary antibodies, including BCRP (MAB4155), and GAPDH (MA5-15738), along with the HRP-conjugated secondary antibody (31,430), were sourced from Thermo Fisher Scientific (Waltham, MA). Protein bands were detected with an enhanced chemiluminescence (ECL) kit (Thermo Fisher Scientific, Waltham, MA). Band intensities were analyzed using ImageJ software (NIH, MD), with protein expression levels normalized to GAPDH as the loading control before statistical evaluation (Chen et al., 2024).

### 2.5 Immunofluorescence assay

The NCI-H460 and NCI-H460/TPT10 cell lines were seeded into a sterile 24-well plate at a concentration of 1 × 10^5^ cells per well. Following a 24-h incubation period that allowed for cellular stabilization and adherence to the glass coverslip, a subsequent 72-h exposure to either a vehicle control or 3 μM of tinodasertib was implemented. Subsequently, the cells were processed for analysis and subjected to immunofluorescence staining, followed by imaging carried out using previously described methods (Lei et al., 2024).

### 2.6 [^3^H]-mitoxantrone accumulation and efflux assay

[^3^H]-Mitoxantrone was employed in both accumulation and efflux assays as outlined previously (Cui et al., 2019). NCI-H460 and NCI-H460/TPT10 cells were seeded at a uniform density of 1 × 10^5^ cells per well in 24-well plates. After incubating overnight, tinodasertib (3 μM) or Ko143 (3 μM) was added to specific wells 2 hours before treating the cells with [^3^H]-mitoxantrone. Following a 2-h exposure to [^3^H]-mitoxantrone, the medium was replaced with fresh medium containing the reversal agent. At designated intervals (0, 30, and 60 min), cells were washed, lysed, and transferred to vials containing 5 mL of scintillation fluid. Radioactivity in each sample was quantified using a scintillation analyzer from Packard Instrument Company, Inc. (Downers Grove, IL, United States).

### 2.7 ATPase assay

The ATPase activity associated with ABCG2 was evaluated in the presence of tinodasertib at varying concentrations (0–40 μM) following a previously described protocol (Wu et al., 2020). In summary, ABCG2-containing membrane vesicles were incubated in assay buffer with or without sodium orthovanadate. Different concentrations of tinodasertib were added, and the mixture was maintained at 37°C for 3 min. The hydrolysis of ATP was triggered by introducing 5 mM ATP, and the reaction was halted by adding an SDS solution. The inorganic phosphate generated was measured using a colorimetric approach (Wu et al., 2021b).

### 2.8 Anti-cancer efficacy test in 3D multicellular tumor spheroids (MCTSs)

NCI-H460 and NCI-H460/TPT10 cells were plated at a density of 500 cells per well into 96-well plates that had been pre-coated with 1% agarose. The multicellular tumor spheroids (MCTSs) were treated with 100 and 300 nM of tinodasertib for 48 h after seeding, once the spheroid aggregates reached a diameter of approximately 300–400 µm. Imaging and measurements were conducted at 0, 24, 48, 72, 96, 120 h, following previously established protocols (Lei et al., 2024).

### 2.9 Molecular modeling of human ABCG2 and docking of tinodasertib

Molecular modeling was performed with Maestro 11.5 software (Schrodinger, New York), following methods outlined in a previous study. (Cai et al., 2019). The human ABCG2 protein (PDB ID: 6FFC) (Jackson et al., 2018) and the ligand were prepared, and a grid was created within the ABCG2 binding site. Glide XP docking and induced-fit docking were then conducted in accordance with the established procedure.

### 2.10 Statistical analysis

The data are expressed as mean ± SD. Efflux assay results were analyzed using two-way ANOVA, whereas other data were assessed with one-way ANOVA, utilizing GraphPad Prism 10.2.0 software (GraphPad, San Diego, CA, United States). All experiments were repeated a minimum of three times, and differences were considered statistically significant when p < 0.05.

## 3 Results and discussion

### 3.1 Tinodasertib increased the sensitivity of ABCG2-overexpressing cell lines to anticancer drugs

**FIGURE 1:**
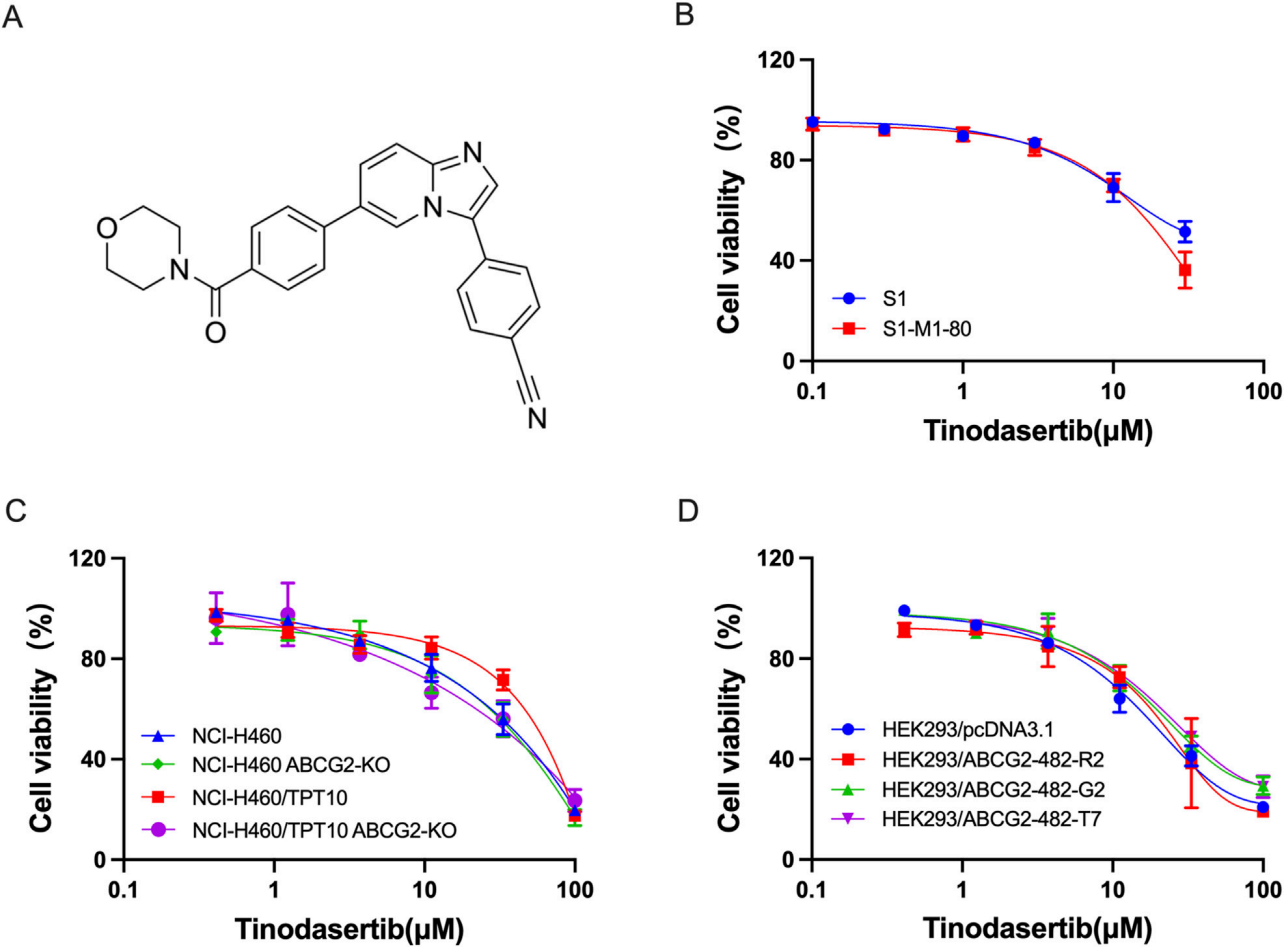
The impact of tinodasertib on the survival of both parental and ABCG2-overexpressing cell lines. **(A)** Tinodasertib’s chemical structure. **(B)** Viability curves for colon cancer cell lines S1 and S1-M1-80. **(C)** Viability curves for non-small cell lung cancer lines, including NCI-H460 and NCI-H460/TPT10, as well as NCI-H460 ABCG2-KO and NCI-H460/TPT10 ABCG2-KO cancer cells **(D)** Viability curves for HEK293 cells and HEK293/ABCG2-overexpressing cells.

This research investigated the ability of tinodasertib (chemical structure depicted in Figure 1A) to reverse MDR in cancer cells and HEK293 cells that overexpress the ABCG2 transporter. To determine concentrations of tinodasertib that would minimally impact cell viability, its toxicity was first tested in the cell lines included in the study. As shown in Figures 1B–D, the cytotoxicity of tinodasertib was not affected by the expression level of ABCG2. Concentrations lower than the IC20 value after a 72-h incubation period were chosen. Based on these findings, further experiments were conducted using tinodasertib at concentrations of 0.3, 1, and 3 μM.

Table 1 demonstrates that S1-M1-80 colon cancer cells displayed significant resistance to mitoxantrone (around 104-fold) and topotecan (approximately 43-fold) compared to the parental S1 cells. When treated with combinations including tinodasertib at 0.3, 1, and 3 μM, the drug’s cytotoxicity was markedly enhanced, and resistance levels were significantly reduced. Specifically, mitoxantrone resistance decreased to 49-, 38-, and 8-fold, while topotecan resistance dropped to 19-, 13-, and 8-fold in the resistant S1-M1-80 cells. These treatments had minimal effects on the parental S1 cells. Additionally, the reversal effect observed with tinodasertib at 3 µM was comparable to that of KO143, a reference ABCG2 inhibitor.

**TABLE 1:** The reversal effect of Tinodasertib in colon cancer cells overexpressing the ABCG2 transporter.

Treatment	IC_50_ value ± SD^a^ (μM, resistance fold^b^)	
	S1	S1-M1-80
Mitoxantrone	0.043 ± 0.010 (1.00)	4.483 ± 0.497 (103.59)
+ Tinodasertib 0.3 μM	0.044 ± 0.010 (1.02)	2.123 ± 0.739 (49.05)^*^
+ Tinodasertib 1 μM	0.043 ± 0.007 (0.99)	1.657 ± 0.524 (38.29)^*^
+ Tinodasertib 3 μM	0.035 ± 0.002 (0.82)	0.346 ± 0.179 (8.00)^*^
+ Ko143 3 μM	0.040 ± 0.003 (0.92)	0.126 ± 0.076 (2.91)^*^
Topotecan	0.559 ± 0.036 (1.00)	23.837 ± 0.215 (42.66)
+ Tinodasertib 0.3 μM	0.454 ± 0.016 (0.81)	10.477 ± 0.079 (18.75)^*^
+ Tinodasertib 1 μM	0.615 ± 0.018 (1.10)	7.145 ± 0.032 (12.79)^*^
+ Tinodasertib 3 μM	0.458 ± 0.012 (0.82)	4.655 ± 0.085 (8.33)^*^
+ Ko143 3 μM	0.504 ± 0.015 (0.90)	2.733 ± 0.047 (4.89)^*^
Cisplatin	4.265 ± 0.223 (1.00)	3.996 ± 0.402 (0.94)
+ Tinodasertib 0.3 μM	3.542 ± 0.630 (0.83)	4.014 ± 0.163 (0.94)
+ Tinodasertib 1 μM	3.725 ± 0.428 (0.87)	3.816 ± 0.727 (0.89)
+ Tinodasertib 3 μM	3.309 ± 0.315 (0.78)	4.138 ± 0.626 (0.97)
+ Ko143 3 μM	3.606 ± 0.271 (0.85)	3.696 ± 0.561 (0.87)

^a^ The IC50 values are expressed as the mean ± standard deviation (SD) based on a minimum of three independent experiments, each conducted in triplicate.

^b^ The resistance fold (Rf) was determined by dividing the IC50 of the substrates, with or without the inhibitor, by the IC50 of the parental cells in the absence of a reversal agent.

*P < 0.05 compared to the control group without a reversal agent.

The NCI-H460/TPT10 lung cancer cells exhibit substantial resistance to mitoxantrone (approximately 114-fold) and topotecan (around 166-fold) in comparison to the parental NCI-H460 cells (Table 2). The combination treatment with tinodasertib at concentrations of 0.3, 1, and 3 µM notably increased cytotoxicity, reducing the resistance fold of mitoxantrone to 31-, 14-, and 12-fold, and topotecan to 73-, 37-, and 17-fold, respectively, in the drug-resistant cells, while having minimal influence on the parental cells. The reversal effect of tinodasertib at 3 µM was comparable to that of Ko143, a known ABCG2 inhibitor. To investigate whether tinodasertib’s reversal effect is associated with inhibiting ABCG2 activity, combinational treatments were conducted in ABCG2 gene-knockout cells, specifically NCI-H460ABCG2 KO and NCI-H460/TPT10ABCG2-KO. As indicated in Table 3, the deletion of the ABCG2 gene in drug-resistant NCI-H460/TPT10 cells led to increased sensitivity to mitoxantrone and topotecan. Importantly, tinodasertib’s MDR reversal effect was negligible in the ABCG2-knockout cells, suggesting that this effect relies on the presence of the ABCG2 transporter.

**TABLE 2:** The reversal effect of tinodasertib in lung cancer cells overexpressing the ABCG2 transporter.

Treatment	IC_50_ value ± SD^a^ (μM, resistance fold^b^)	
	NCI-H460	NCI-H460/TPT10
Mitoxantrone	0.015 ± 0.007 (1.00)	1.674 ± 0.062 (114.14)
+ Tinodasertib 0.3 μM	0.018 ± 0.009 (1.21)	0.449 ± 0.179 (30.60)^*^
+ Tinodasertib 1 μM	0.016 ± 0.008 (1.06)	0.208 ± 0.082 (14.20)^*^
+ Tinodasertib 3 μM	0.013 ± 0.007 (0.91)	0.171 ± 0.069 (11.64)^*^
+ Ko143 3 μM	0.012 ± 0.004 (0.84)	0.078 ± 0.052 (5.34)^*^
Topotecan	0.054 ± 0.008 (1.00)	8.992 ± 0.738 (165.58)
+ Tinodasertib 0.3 μM	0.044 ± 0.008 (0.81)	3.965 ± 0.989 (73.01)^*^
+ Tinodasertib 1 μM	0.063 ± 0.014 (1.17)	2.021 ± 0.238 (37.21)^*^
+ Tinodasertib 3 μM	0.049 ± 0.005 (0.91)	0.935 ± 0.516 (17.22)^*^
+ Ko143 3 μM	0.044 ± 0.011 (0.81)	0.341 ± 0.086 (6.28)^*^
Cisplatin	1.301 ± 0.171 (1.00)	1.492 ± 0.082 (1.15)
+ Tinodasertib 0.3 μM	1.323 ± 0.215 (1.02)	1.496 ± 0.095 (1.15)
+ Tinodasertib 1 μM	1.197 ± 0.287 (0.92)	1.388 ± 0.115 (1.03)
+ Tinodasertib 3 μM	1.192 ± 0.150 (0.92)	1.500 ± 0.106 (1.15)
+ Ko143 3 μM	1.364 ± 0.213 (1.05)	1.328 ± 0.343 (1.02)

^a^ The IC50 values are expressed as the mean ± standard deviation (SD) based on a minimum of three independent experiments, each conducted in triplicate.

^b^ The resistance fold (Rf) was determined by dividing the IC50 of the substrates, with or without the inhibitor, by the IC50 of the parental cells in the absence of a reversal agent.

*P < 0.05 compared to the control group without a reversal agent.

**TABLE 3:** The reversal effect of Tinodasertib in lung cancer cells without ABCG2 expression.

Treatment	IC_50_ value ± SD^a^ (μM, resistance fold^b^)	
	NCI-H460 ABCG2-KO	NCI-H460/TPT10 ABCG2-KO
Mitoxantrone	0.144 ± 0.010 (1.00)	0.107 ± 0.006 (0.75)
+ Tinodasertib 0.3 μM	0.137 ± 0.021 (0.95)	0.125 ± 0.024 (0.87)
+ Tinodasertib 1 μM	0.096 ± 0.020 (0.67)	0.093 ± 0.023 (0.65)
+ Tinodasertib 3 μM	0.102 ± 0.045 (0.71)	0.099 ± 0.004 (0.69)
+ Ko143 3 μM	0.094 ± 0.032 (0.65)	0.127 ± 0.037 (0.88)
Topotecan	0.035 ± 0.006 (1.00)	0.045 ± 0.007 (1.28)
+ Tinodasertib 0.3 μM	0.034 ± 0.011 (0.97)	0.033 ± 0.004 (0.93)
+ Tinodasertib 1 μM	0.031 ± 0.004 (0.88)	0.032 ± 0.006 (0.91)
+ Tinodasertib 3 μM	0.022 ± 0.001 (0.61)	0.030 ± 0.006 (0.85)
+ Ko143 3 μM	0.028 ± 0.008 (0.79)	0.027 ± 0.003 (0.76)
Cisplatin	1.601 ± 0.081 (1.00)	1.437 ± 0.073 (0.90)
+ Tinodasertib 0.3 μM	1.706 ± 0.047 (1.07)	1.293 ± 0.006 (0.81)
+ Tinodasertib 1 μM	1.814 ± 0.091 (1.13)	1.383 ± 0.066 (0.86)
+ Tinodasertib 3 μM	1.720 ± 0.127 (1.07)	1.164 ± 0.106 (0.73)
+ Ko143 3 μM	1.816 ± 0.015 (1.13)	1.381 ± 0.032 (0.86)

^a^ The IC50 values are expressed as the mean ± standard deviation (SD) based on a minimum of three independent experiments, each conducted in triplicate.

^b^ The resistance fold (Rf) was determined by dividing the IC50 of the substrates, with or without the inhibitor, by the IC50 of the parental cells in the absence of a reversal agent.

*P < 0.05 compared to the control group without a reversal agent.

Our findings demonstrate that Tinodasertib effectively antagonizes ABCG2-mediated MDR at clinically achievable concentrations, suggesting its potential as an inhibitor of ABCG2. Combining tinodasertib with chemotherapeutic agents that are ABCG2 substrates may benefit a subset of cancer patients with MDR tumors expressing the ABCG2 transporter.

To further investigate tinodasertib’s reversal effect, we examined its impact in HEK293 cells overexpressing various ABCG2 variants, including HEK293/pcDNA3.1, HEK293/ABCG2-482-R2, HEK293/ABCG2-482-G2, and HEK293/ABCG2-482-T7. Mutations at the R482 position of ABCG2 can alter substrate recognition and transport efficiency (Gose et al., 2024; Homolya, 2021; Manolaridis et al., 2018). And some inhibitors exhibit selective reversal effects on specific ABCG2 mutants (Kuhnert et al., 2024; Valdameri et al., 2023; Krapf et al., 2018).As shown in Table 4, tinodasertib enhanced sensitivity in HEK293/ABCG2-482-G2, HEK293/ABCG2-482-R2, and HEK293/ABCG2-482-T7 cells, similar to its effects in drug-resistant cancer cells, suggesting that R482 mutations do not compromise its MDR reversal activity. Additionally, tinodasertib did not affect the IC50 of cisplatin, a non-ABCG2 substrate, in HEK293/pcDNA3.1 or drug-resistant cells, confirming that its MDR reversal effect is specific to ABCG2.

**TABLE 4:** The reversal effect of Tinodasertib in gene-transfected HEK293 cells overexpressing the ABCG2 transporter.

Treatment	IC_50_ value ± SD^a^ (μM, Resistance-fold^b^)			
	pcDNA3.1	ABCG2-G2	ABCG2-R2	ABCG2-T7
Topotecan	0.101 ± 0.012 (1.00)	1.666 ± 0.093 (16.51)	2.130 ± 0.074 (21.11)	1.498 ± 0.140 (14.85)
+ Tinodasertib 0.3 μM	0.110 ± 0.011 (1.09)	1.015 ± 0.029 (10.06)^*^	0.749 ± 0.026 (7.42)^*^	0.709 ± 0.139 (7.02)^*^
+ Tinodasertib 1 μM	0.124 ± 0.014 (1.23)	0.569 ± 0.097 (5.64)^*^	0.552 ± 0.075 (5.47)^*^	0.338 ± 0.079 (3.35)^*^
+ Tinodasertib 3 μM	0.086 ± 0.015 (0.85)	0.365 ± 0.070 (3.62)^*^	0.293 ± 0.026 (2.90)^*^	0.314 ± 0.032 (3.11)^*^
+ Ko143 3 μM	0.091 ± 0.031 (0.90)	0.352 ± 0.048 (3.49)^*^	0.173 ± 0.083 (1.72)^*^	0.300 ± 0.056 (2.98)^*^
Cisplatin	5.512 ± 0.131 (1.00)	5.234 ± 0.308 (0.95)	4.079 ± 0.324 (0.74)	5.449 ± 0.341 (0.99)
+ Tinodasertib 3 μM	4.949 ± 0.665 (0.90)	5.358 ± 0.368 (0.97)	4.126 ± 0.349 (0.75)	6.961 ± 0.199 (1.26)
+ Ko143 3 μM	4.884 ± 0.809 (0.89)	5.016 ± 0.308 (0.91)	4.412 ± 0.002 (0.80)	4.615 ± 0.103 (0.84)

^a^ The IC50 values are expressed as the mean ± standard deviation (SD) based on a minimum of three independent experiments, each conducted in triplicate.

^b^ The resistance fold (Rf) was determined by dividing the IC50 of the substrates, with or without the inhibitor, by the IC50 of the parental cells in the absence of a reversal agent.

*P < 0.05 compared to the control group without a reversal agent.

### 3.2 Tinodasertib showed no impact on the protein expression levels of ABCG2 transporters

**FIGURE 2:**
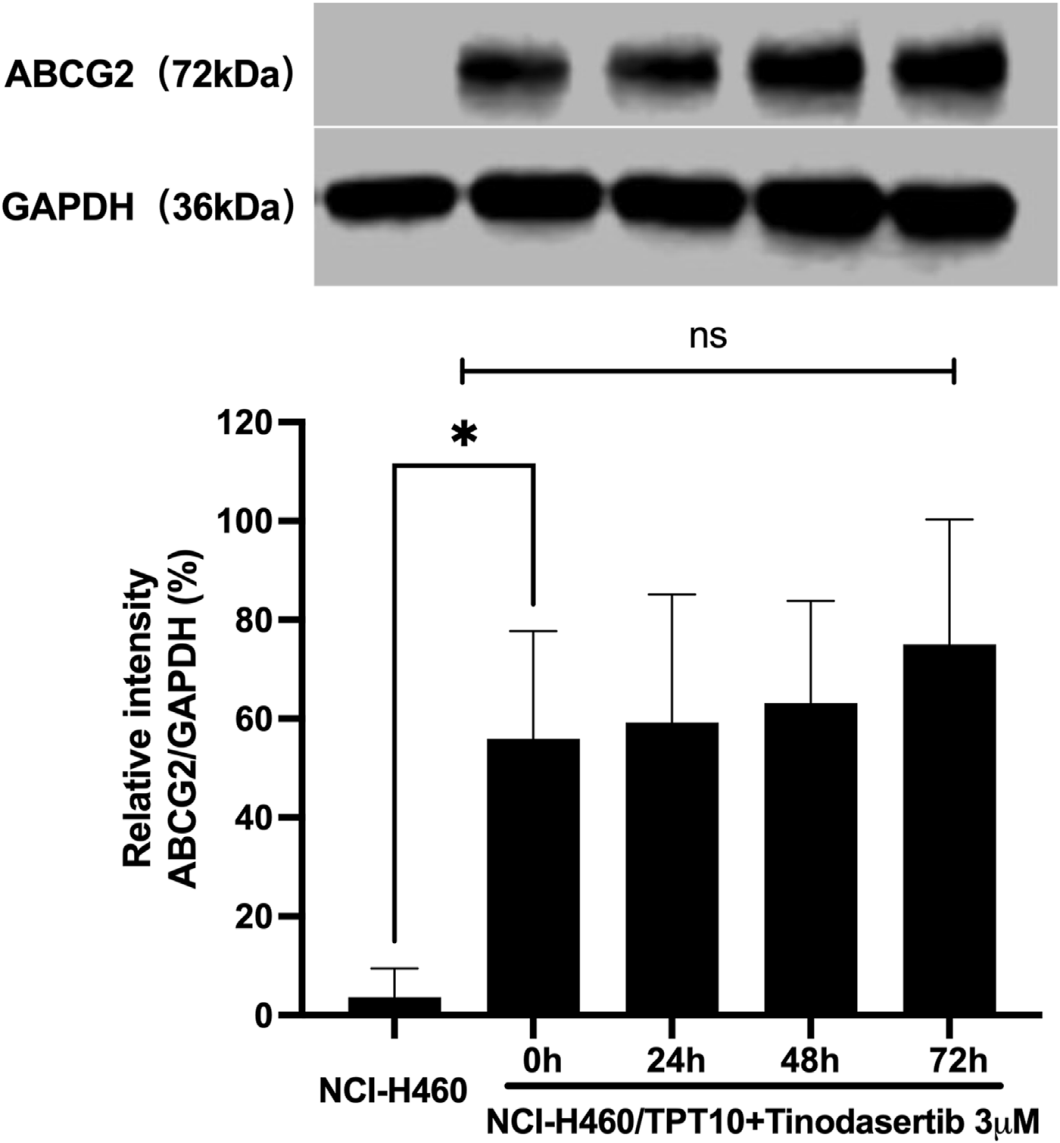
Tinodasertib showed no impact on the protein expression levels of ABCG2 transporters.The detection and relative intensity of ABCG2 expression in NCI-H460/TPT10 cells treated with 3 μM tinodasertib over the specified time points are presented as mean ± SD and are representative of three independent experiments. *P < 0.05 compared to the control group (NCI-H460).

Given its ability to counteract ABCG2-mediated MDR, additional experiments were performed to investigate the underlying mechanisms of its MDR reversal effect. As illustrated in Figure 2, when NCI-H460/TPT10 cells were treated with 3 μM tinodasertib for 0, 24, 48, and 72 h, no significant changes were observed in the expression level of the ABCG2 protein (72 kDa) within this ABCG2-overexpressing cell line. This suggests that tinodasertib does not influence the expression of the ABCG2 transporter.

### 3.3 Tinodasertib had no impact on the subcellular localization of ABCG2 transporters

**FIGURE 3:**
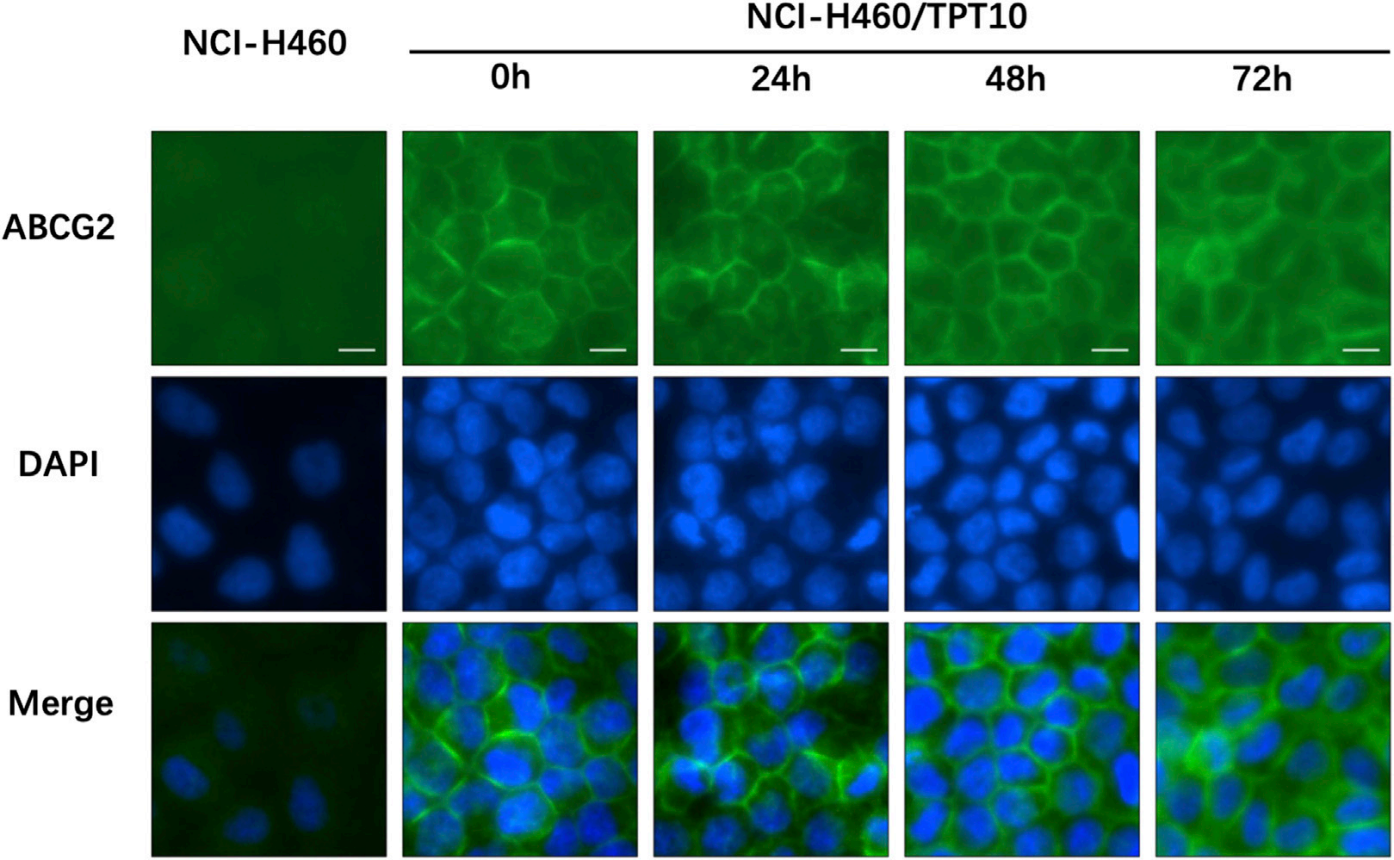
Tinodasertib treatment had no impact on the subcellular localization of ABCG2 transporters in the NCI-H460/TPT10 cell line overexpressing ABCG2. The subcellular distribution of ABCG2 in NCI-H460/TPT10 cells treated with 3 μM tinodasertib for 0, 24, 48, and 72 h is shown. ABCG2 is visualized in blue, while DAPI was used to counterstain the nuclei. NCI-H460 cells served as the control group. Scale bar: 100 μm.

To investigate the subcellular localization of the ABCG2 transporter, immunofluorescence analysis was conducted on NCI-H460/TPT10 cells treated with 3 μM tinodasertib for 0, 24, 48, and 72 h. As depicted in Figure 3, the ABCG2 transporter was primarily situated on the plasma membrane under all tested conditions. Treatment with tinodasertib did not lead to any notable changes in the subcellular distribution of ABCG2, suggesting that the drug does not influence the trafficking or localization of the ABCG2 protein.

### 3.4 Tinodasertib enhances drug accumulation and suppresses ABCG2-mediated efflux activity in cancer cell lines that overexpress ABCG2

**FIGURE 4:**
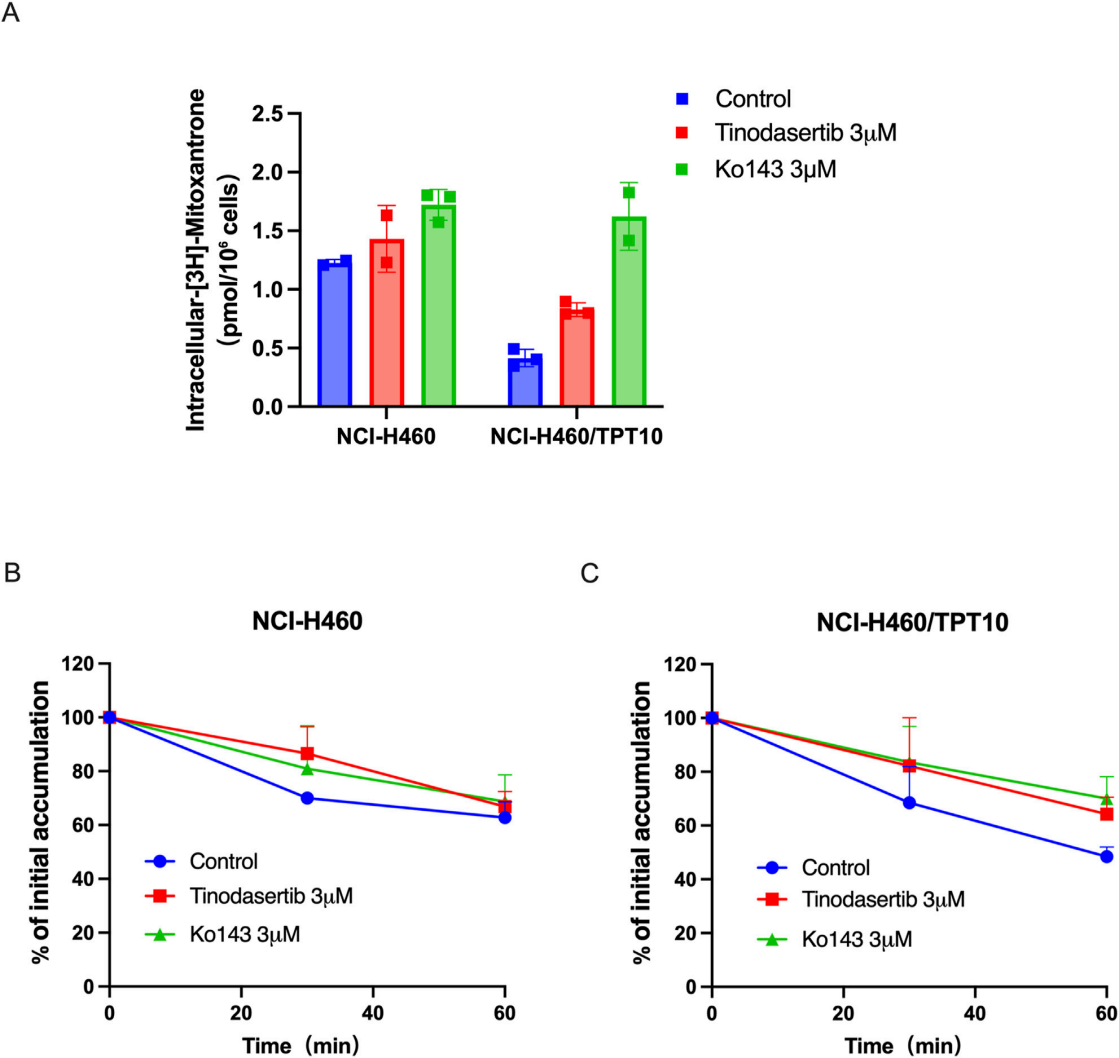
Tinodasertib enhances drug accumulation and suppresses ABCG2-mediated efflux activity in cancer cell lines that overexpress ABCG2. **(A)** The impact of tinodasertib on [^3^H]-mitoxantrone accumulation was examined in NCI-H460 and NCI-H460/TPT10 cell lines. Ko143 (3 μM) served as a positive control for cells overexpressing ABCG2. **(B, C)** The impact of tinodasertib on [^3^H]-mitoxantrone efflux was assessed in NCI-H460 (B) and NCI-H460/TPT10 (C) cell lines. The data, presented as mean ± SD, represent the outcomes of three separate experiments. *p < 0.05 compared to the control group.

Tinodasertib has been shown to effectively counteract ABCG2-mediated MDR without altering the protein’s expression or subcellular distribution. To elucidate the mechanism behind this phenomenon, drug accumulation and efflux assays were performed using NCI-H460/TPT10 cells, which overexpress ABCG2. In NCI-H460/TPT10 cells, the intracellular concentration of [^3^H]-mitoxantrone was significantly increased in cells exposed to 3 μM tinodasertib compared to untreated controls, as shown in Figure 4A. In contrast, the parental NCI-H460 cells, which lack ABCG2 overexpression, exhibited no notable changes in drug accumulation.

Further analysis revealed that tinodasertib effectively inhibited ABCG2’s drug efflux activity. Efflux assays demonstrated a substantial reduction in [^3^H]-mitoxantrone efflux from ABCG2-overexpressing cells treated with vehicle control (Figure 4B). When treated with tinodasertib, the ABCG2-overexpressing cells retained higher intracellular drug levels, particularly within the first 60 min, compared to the rapid decline observed in vehicle treated cells. These findings indicate that tinodasertib enhances the intracellular retention of anticancer drugs by inhibiting the efflux activity of the ABCG2 transporter, thus overcoming MDR at a molecular level.

### 3.5 Tinodasertib suppressed the ABCG2-associated ATPase activity

**FIGURE 5:**
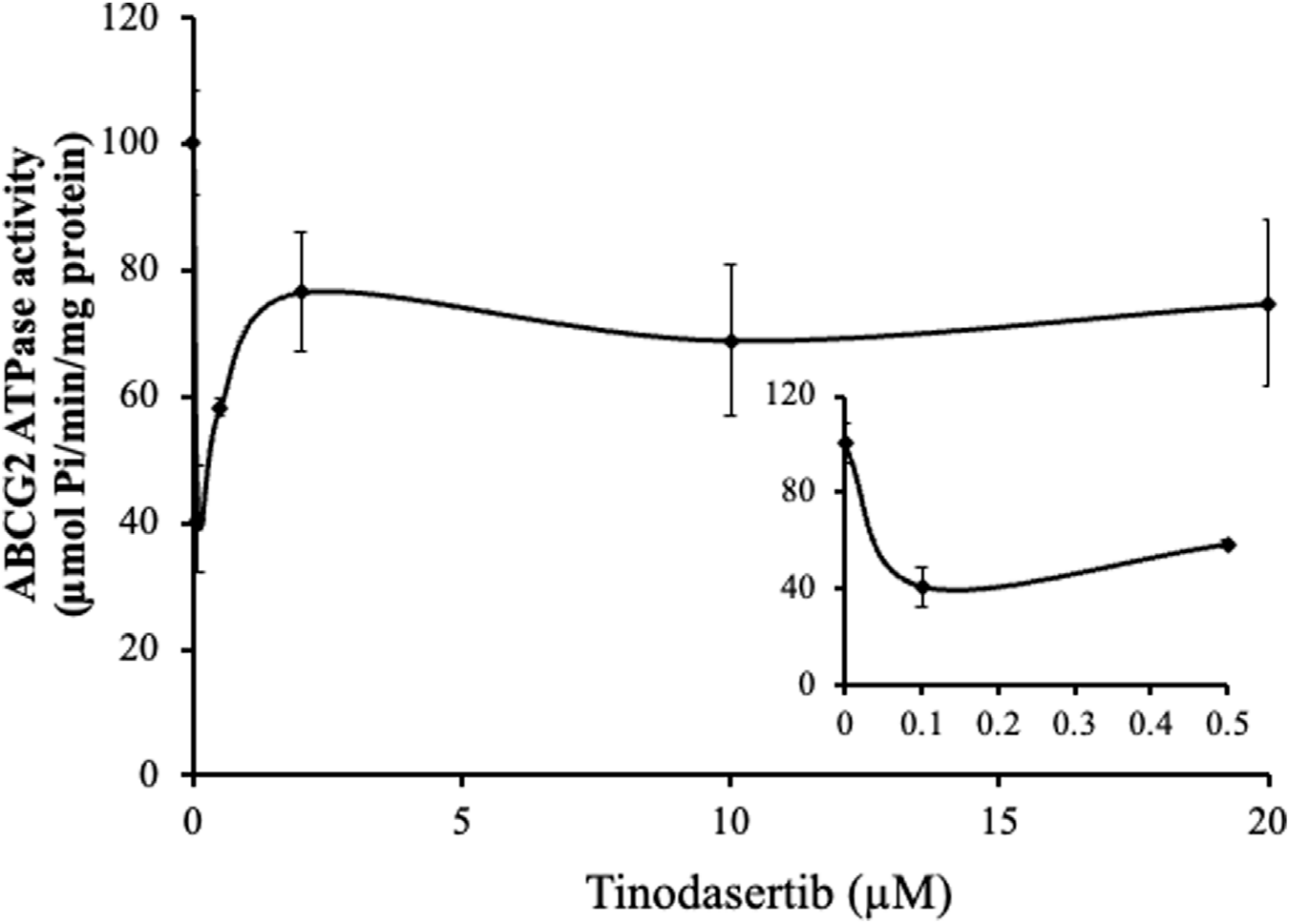
Tinodasertib suppressed the ABCG2-associated ATPase activity. The effect of varying tinodasertib concentrations on ABCG2 ATPase activity is illustrated. The inset graphs highlight the influence of 0–20 μM tinodasertib on ATPase activity associated with ABCG2. Results are expressed as mean ± SD and are representative of three separate experiments.

Our *in vitro* ATPase assays demonstrated that tinodasertib modulated the basal ATPase activity of the ABCG2 transporter in a concentration-dependent manner (0–20 μM), as shown in Figure 5. The results revealed that tinodasertib primarily inhibited the basal ATPase activity rather than stimulating it. These findings suggest that tinodasertib interacts with the ABCG2 transporter, potentially inhibiting its ATP hydrolysis activity. It is also possible that tinodasertib competes directly with ATP for binding at the ATPase site rather than primarily targeting the drug-substrate binding site.

### 3.6 Targeted toxicity of tinodasertib in lung cancer MCTSs with overexpression of ABCB2

**FIGURE 6:**
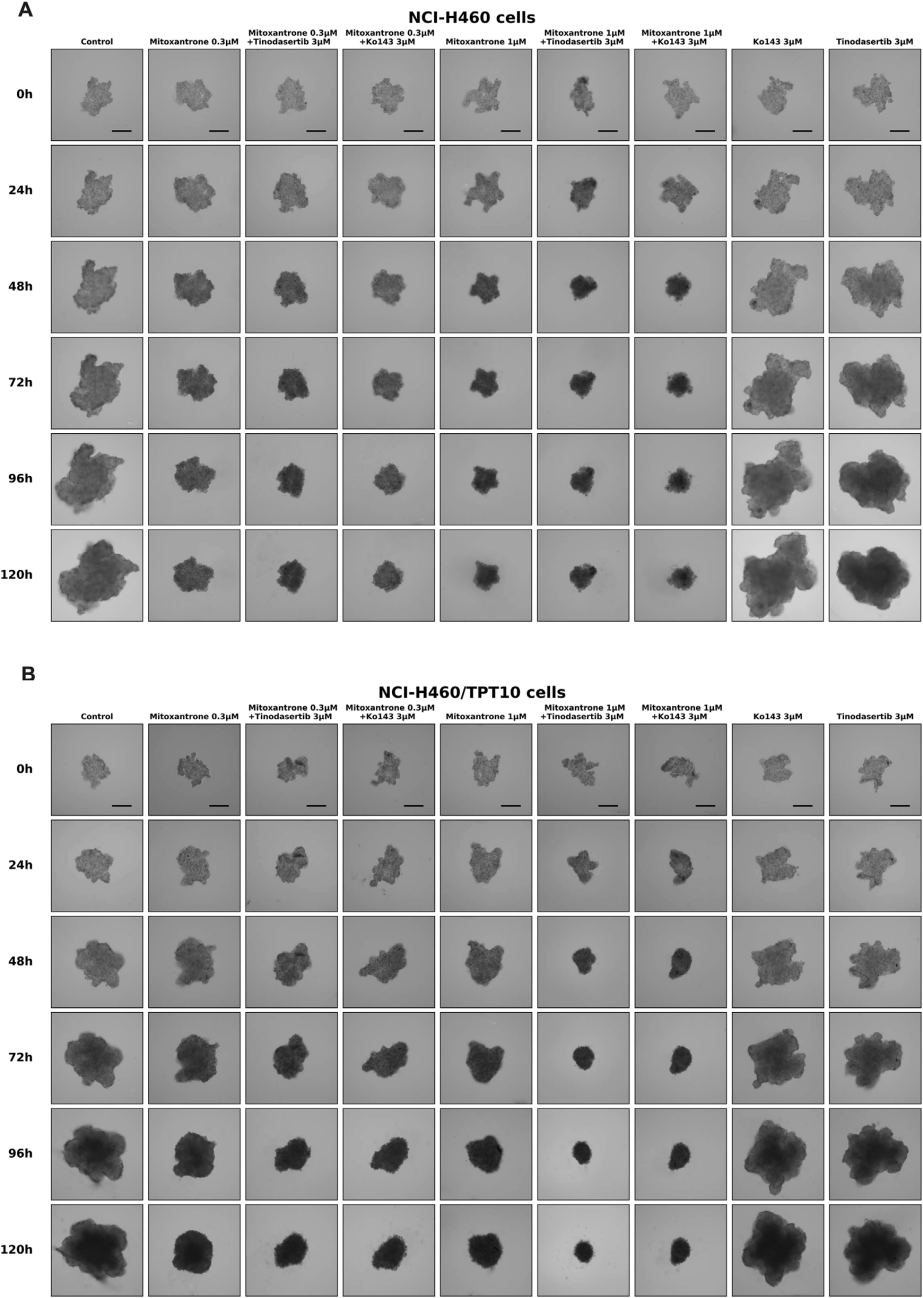
Sample images of multicellular tumor spheroids (MCTSs) derived from NCI-H460 and NCI-H460/TPT10 cells under various treatment conditions. **(A)** Representative images of MCTSs from NCI-H460 cells. **(B)** Representative images of MCTSs from NCI-H460/TPT10 cells. Evaluations were conducted at 0, 24, 48, 72, 96, and 120 h, with scale bars indicating 250 μm.

The experimental images demonstrate the effects of various treatments on the growth of NCI-H460 and NCI-H460/TPT10 cells over 120 h (Figures 6A,B). Mitoxantrone, at 0.3 μM and 1 μM, effectively inhibited the cell growth of parental NCI-H460 cells but not drug-resistant NCI-H460/TPT10 cells compared to the control, with stronger effects at the higher concentration. The combination of mitoxantrone with tinodasertib (T, 3 μM) or Ko143 (3 μM) further suppressed cell growth in drug-resistant NCI-H460/TPT10 cells, indicating potential synergistic effects. In both cell lines, single-agent treatments with tinodasertib or Ko143 showed minimal inhibitory effects. These results suggest that mitoxantrone has significant anti-cancer activity, which can be enhanced through combination with ABCG2 inhibitors tinodasertib or Ko143, highlighting the potential of combination therapies to improve therapeutic efficacy.

### 3.7 Docking analysis of the binding of tinodasertib with human ABCG2 model

**FIGURE 7:**
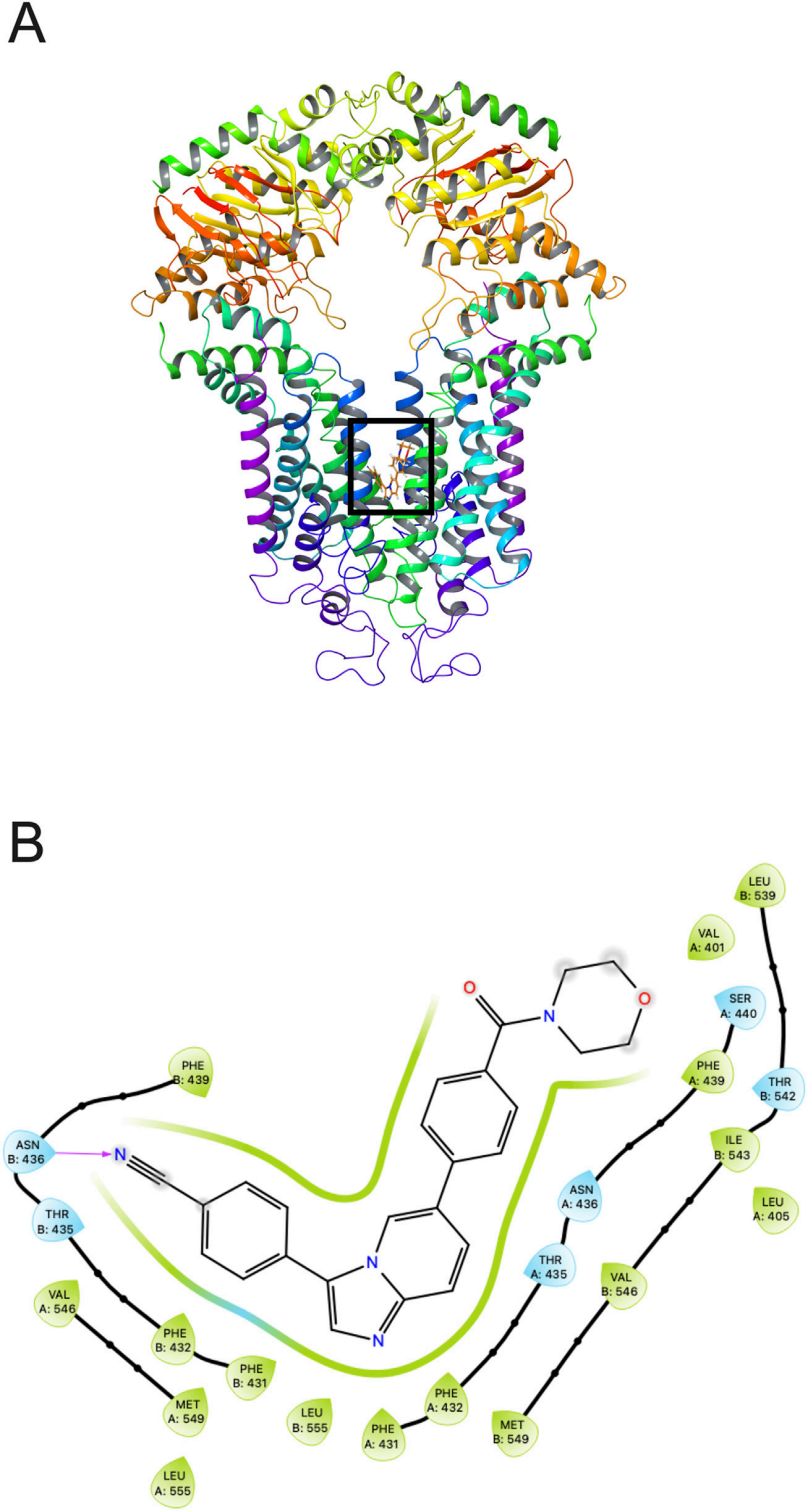
Tinodasertib’s interaction with the human ABCG2 protein (PDB ID: 6ETI). **(A)** The highest-scoring pose of tinodasertib within the drug-binding pocket of the ABCG2 protein is depicted. The ABCG2 structure is shown as tubes and ribbons in various colors, while tinodasertib is illustrated as colored sticks (carbon: grey; oxygen: red; nitrogen: blue). **(B)** A two-dimensional representation illustrates the interactions between tinodasertib and the drug-binding pocket of ABCG2. Significant residues are depicted as color-coded bubbles, with hydrophobic residues in green and polar ones in blue. Critical hydrogen bonds are indicated by purple dashed lines, while π-π stacking interactions are marked with green lines. Direct interactions between tinodasertib and residues like ASN436 and THR435 emphasize its binding mechanism.

Our docking simulations (Figure 7) revealed that tinodasertib effectively binds to the ATP binding site at the nucleotide-binding domain (NBD) of the human ABCG2 crystal structure (PDB ID: 6ETI) (Jackson et al., 2018), with a notable docking score of −10.568 kcal/mol. This highlights the potential of targeting this site for further research. The binding of tinodasertib is supported by hydrophobic interactions with key amino acid residues, such as Leu405, Val401, Thr542, Leu539, and Ile543 in chain A, and Phe439, Asn436, and Thr435 in chain B. Furthermore, a hydrogen bond is established between Asn436 and a nitrogen atom in tinodasertib, complemented by a π-π stacking interaction involving Phe439 and the compound’s aromatic rings. These interactions anchor tinodasertib securely within the hydrophobic pocket of ABCG2, indicating its potential to block the transporter and improve the effectiveness of anticancer drugs like mitoxantrone, SN-38, and topotecan by inhibiting their efflux. However, additional experiments are required to validate these results.

## 4 Discussion

Overcoming MDR remains a major obstacle in cancer treatment, especially when resistance is driven by ABC transporters such as ABCG2 (Zhu et al., 2024; Koirala and DiPaola, 2024; Li et al., 2024a; To et al., 2024). As a potential solution, combining chemotherapeutic agents with reversal compounds that inhibit ABC transporter activity presents a promising approach to enhance the efficacy of chemotherapy in cancer patients (Li et al., 2024b; Kuhnert et al., 2024; Fu et al., 2024). This study focused on evaluating the ability of tinodasertib to counteract ABCG2-mediated drug resistance and investigated its underlying mechanisms through various experimental methods.

MTT assays were performed to identify the concentrations of tinodasertib that were relatively non-toxic to the cells utilized in this study. Based on the cytotoxicity findings, tinodasertib concentrations of 0.3, 1, and 3 μM were selected for the reversal experiments. The results demonstrated that tinodasertib effectively counteracts ABCG2-mediated MDR in various cancer cell lines and HEK293 cells expressing different ABCG2 variants. Tinodasertib significantly enhanced the cytotoxic effects of ABCG2 substrate drugs, including mitoxantrone and topotecan, while showing minimal impact on non-resistant parental cells or on the cytotoxicity of non-ABCG2 substrates, such as cisplatin. The reversal effect of tinodasertib was found to rely on the presence of functional ABCG2, as it was notably reduced in ABCG2 gene-knockout cells. Furthermore, tinodasertib maintained its reversal activity in ABCG2 mutants with R482 residue alterations, demonstrating its broad effectiveness across various ABCG2 variants. These results underscore tinodasertib’s potential as a powerful ABCG2 inhibitor with promising clinical applications for addressing MDR in cancer treatment.

The reversal of MDR mediated by ABC transporters can occur due to decreased expression of ABC transporter proteins or alterations in their subcellular localization (Sunshine et al., 2016; Amawi et al., 2019; Yan et al., 2017). To investigate these mechanisms, we utilized Western blotting and immunofluorescence assays. However, treatment with tinodasertib (3 μM) for up to 72 h did not lead to a significant reduction in the protein levels of ABCB1 or ABCG2 transporters in NCI-H460 or NCI-H460/TPT10 cells. Similarly, incubation with tinodasertib at the same concentration and duration did not cause notable changes in the subcellular localization of ABCG2 transporters in these cells. These results indicate that tinodasertib’s ability to reverse MDR is not linked to alterations in ABC transporter protein levels or their subcellular distribution.

Given that the MDR reversal effects of tinodasertib were not linked to changes in ABC transporter protein levels or subcellular localization, we next investigated its impact on drug accumulation and efflux. Our findings reveal that tinodasertib significantly reduces the efflux of [^3^H]-mitoxantrone in ABCG2-overexpressing cells, effectively inhibiting the drug efflux activity of ABCG2. Treated cells displayed substantially higher drug retention levels compared to untreated controls, particularly within the first 60 min, where retention remained close to initial concentrations. In contrast, untreated cells experienced a rapid decline in drug retention. These results indicate that tinodasertib enhances the intracellular accumulation of anticancer drugs by blocking ABCG2-mediated efflux. Given the pivotal role of ABCG2 in protecting cancer cells from chemotherapeutic agents by actively exporting drugs, the ability of tinodasertib to inhibit this process suggests a promising strategy to sensitize drug-resistant cells to therapy. Furthermore, these findings support the broader notion that targeting ABCG2 not only improves drug retention but may also restore drug efficacy, particularly for substrates of this transporter (Xu et al., 2020; Di Micco et al., 2022; Ma et al., 2022). This underscores the potential of tinodasertib as a valuable adjunct in combination chemotherapy to overcome multidrug resistance and improve clinical outcomes for cancer patients.

The activity of ABC transporters is powered by ATP hydrolysis, a process that can be modulated by particular substrates or inhibitors (Banerjee et al., 2023; Clouser and Atkins, 2022; Xing et al., 2020; Thomas and Tampé, 2020). To explore the mechanism behind tinodasertib’s interaction with ABCG2, we examined its effects on the transporter’s ATPase activity. Results from our ATPase assays indicated that tinodasertib influences the basal ATPase activity of ABCG2 in a concentration-dependent manner, predominantly suppressing its function. These findings suggest that tinodasertib interacts directly with ABCG2, potentially binding to its drug-substrate site and suppressing its ATP hydrolysis function. Similar ATPase-inhibitory effects have been reported for other ABCG2 inhibitors, such Venetoclax (Wang et al., 2020), Sitravatinib (Yang et al., 2020), and BMS-599626 (Ashar et al., 2020), suggesting that tinodasertib may share a comparable mechanism of action through interference with ABCG2’s ATPase activity. This interaction highlights a possible mechanism by which tinodasertib inhibits ABCG2-mediated drug efflux, further supporting its role as an effective reversal agent for multidrug resistance in cancer therapy.

MCTSs provide a more physiologically relevant model for studying drug efficacy and resistance mechanisms compared to traditional monolayer cultures (Bess et al., 2024; Wang et al., 2024; Kulesza et al., 2023; Nayak et al., 2023). In this study, we evaluated the selective toxicity of tinodasertib in ABCG2-overexpressing lung cancer MCTSs to better understand its potential as a reversal agent in a three-dimensional tumor microenvironment. Our findings indicate that mitoxantrone demonstrates significant anticancer activity in both NCI-H460 and NCI-H460/TPT10 cells, with greater growth inhibition observed at higher concentrations. Notably, the combination of Mitoxantrone with tinodasertib or Ko143 further enhanced the suppression of cell growth, suggesting synergistic effects. While single-agent treatments with tinodasertib or Ko143 showed moderate efficacy, they were less effective than the combination therapies. These results underscore the potential of combining mitoxantrone with tinodasertib or Ko143 to improve therapeutic outcomes, particularly in overcoming ABCG2-mediated drug resistance.

Molecular docking provides valuable insights into how small molecules interact with specific binding sites on target proteins (González-Ruiz and Gohlke, 2006; Shamim et al., 2024; Moosavi et al., 2022), offering a deeper understanding of the molecular basis for the observed MDR reversal effects of tinodasertib. To further elucidate the interaction between tinodasertib and ABCG2, as well as its potential binding mechanism, docking studies were performed. Docking analysis revealed that tinodasertib exhibits a strong binding affinity for the drug-binding pocket of the ABCG2 transporter (docking score: −10.568). Key interactions, including hydrogen bonding with residues such as ASN436 and THR435 and π-π stacking, were identified, highlighting its binding mechanism. These findings provide structural insights into how tinodasertib interacts with ABCG2, supporting its potential role in modulating ABCG2 activity and contributing to its MDR reversal effects.

## 5 Conclusion

This study provides valuable therapeutic insights by demonstrating that tinodasertib effectively reverses ABCG2-mediated MDR through the inhibition of drug efflux by the ABCG2 transporter. These findings highlight tinodasertib’s potential as a targeted MDR reversal agent and underscore its therapeutic promise in enhancing the efficacy of chemotherapeutic agents. Combining tinodasertib with drugs that serve as substrates for ABCG2 presents a promising approach to tackling MDR in cancer treatment. Tinodasertib is effective in reversing ABCG2-mediated MDR at clinically achievable concentrations, supporting its further development as a combination chemotherapy sensitizer.This strategy holds potential to enhance the effectiveness of therapies against drug-resistant tumors. However, additional research, including clinical trials, is necessary to validate its efficacy as a method for combating MDR in oncology.

## Data Availability

The original contributions presented in the study are included in the article/supplementary material, further inquiries can be directed to the corresponding authors.
