# Correction: Reversal of ABCG2-mediated drug resistance by tinodasertib (ETC-206)

**DOI:** 10.3389/fphar.2025.1648210

**Published:** 2025-08-07

**Authors:** Haigan Yang, Zheshen Li, Zhuoxun Wu, Xiang Chen, Letao Bo, Harsh Patel, Bohan Zhang, Wenjun Xiong, Wei Wang, Zhe-Sheng Chen

**Affiliations:** ^1^ Department of Pharmaceutical Sciences, College of Pharmacy and Health Sciences, St. John’s University, Queens, NY, United States; ^2^ The First Affiliated Hospital of Guangzhou University of Chinese Medicine, Guangzhou, Guangdong, China

**Keywords:** ATP-binding cassette (ABC) transporter, tinodasertib, ETC-206, ABCG2, multidrug resistance (MDR)

An incorrect Funding statement was provided. The funder’s name and number were incorrect. The correct funding statement reads:

The author(s) declare that financial support was received for the research and/or publication of this article. This research was supported by the Scientific Research Project of the Guangdong Provincial Administration of Traditional Chinese Medicine (Grant No. 20231125).

The original article has been updated.

